# Current understanding of the genomic abnormities in premature ovarian failure: chance for early diagnosis and management

**DOI:** 10.3389/fmed.2023.1194865

**Published:** 2023-06-02

**Authors:** Xu Yang, Lin Yang

**Affiliations:** ^1^Department of Obstetrics and Gynecology, West China Second University Hospital, Sichuan University, Chengdu, Sichuan, China; ^2^Department of Pharmacy, West China Hospital, Sichuan University, Chengdu, China

**Keywords:** premature ovarian failure (POF), infertility, genomic variants, treatment, clinical trials

## Abstract

Premature ovarian failure (POF) is an insidious cause of female infertility and a devastating condition for women. POF also has a strong familial and heterogeneous genetic background. Management of POF is complicated by the variable etiology and presentation, which are generally characterized by abnormal hormone levels, gene instability and ovarian dysgenesis. To date, abnormal regulation associated with POF has been found in a small number of genes, including autosomal and sex chromosomal genes in folliculogenesis, granulosa cells, and oocytes. Due to the complex genomic contributions, ascertaining the exact causative mechanisms has been challenging in POF, and many pathogenic genomic characteristics have yet to be elucidated. However, emerging research has provided new insights into genomic variation in POF as well as novel etiological factors, pathogenic mechanisms and therapeutic intervention approaches. Meanwhile, scattered studies of transcriptional regulation revealed that ovarian cell function also depends on specific biomarker gene expression, which can influence protein activities, thus causing POF. In this review, we summarized the latest research and issues related to the genomic basis for POF and focused on insights gained from their biological effects and pathogenic mechanisms in POF. The present integrated studies of genomic variants, gene expression and related protein abnormalities were structured to establish the role of etiological genes associated with POF. In addition, we describe the design of some ongoing clinical trials that may suggest safe, feasible and effective approaches to improve the diagnosis and therapy of POF, such as Filgrastim, goserelin, resveratrol, natural plant antitoxin, Kuntai capsule et al. Understanding the candidate genomic characteristics in POF is beneficial for the early diagnosis of POF and provides appropriate methods for prevention and drug treatment. Additional efforts to clarify the POF genetic background are necessary and are beneficial for researchers and clinicians regarding genetic counseling and clinical practice. Taken together, recent genomic explorations have shown great potential to elucidate POF management in women and are stepping from the bench to the bedside.

## Introduction

Premature ovarian failure (POF), also known as premature menopause, causes absent menstruation and infertility before expected female menopause (1). It is characterized by the loss of normal ovarian function, the cessation of menstruation or loss of follicles and the cessation of follicle production (2). Approximately 1% of females are affected by sporadic POF in reproductive years under the age of 40, 1‰ before 30 years old and 0.1% before 20 years old, where familial POF presented the highest incidence frequency of nearly 12.7% (3, 4). Females with POF usually exhibit follicular atresia, hypoestrogenism and ovarian function loss and then present with primary or secondary amenorrhea and infertility (5). Clinically, diagnostic criteria are usually based on follicle-stimulating hormone (FSH) levels in the menopausal range (over 40 IU/L) and estradiol levels (less than 50 pmol/L) in females aged before 40 (6, 7). However, ovarian biopsy and ultrasound have minimal effects in the early diagnosis and screening of POF. The diagnosed POF female will suffer a serious psychosocial, economic and health burden (8). Thus, the prediagnosis of the high-risk POF population becomes particularly important for protection and treatment.

The etiology of POF is highly heterogeneous, with a wide spectrum of causes, such as genetics, living habits, the environment and infection, whereas the genetic contribution is generally considered paramount (9). POF, as one component of genetic disorders, has been widely recognized (10). An increasing number of plausible causative genes have been proposed, including genomic mutation, dysregulated gene expression and signaling cascade functions, whereas the gene framework for interpreting the etiology of POF has yet to be elucidated (11). However, the precise identification of causative genes remains a challenge. Causative genes potentially influence various aspects of the ovary, such as gonadal development, oogenesis, folliculogenesis, meiosis, DNA repair, hormone secretion, metabolism, and immune response (9, 11). Defects in multiple genes cause ovarian failure in animal models. It is reasonable to begin by searching causative genes from different angles related to POF development. With contemporary genetic strategy development, genome-wide association studies (GWASs) and genome-wide sequencing of exomes (WES) approaches provide us with more opportunities to locate susceptible loci and more candidate genes related to POF (12–14).

In particularly, for the early diagnosis of a disease, biomarkers are measurable indicators of a particular disease or physiological state of an organism. In terms of this, any epigenetic phenotype can be attributed to DNA, RNA, or protein changes, which in turn are biomarkers for a specific phenotype (15). The ideal biomarker should be easily detected in blood or primary tissues and meet regulatory approval for the test to be used to make clinically valid decisions (16). Understanding specific POF genetic mechanisms will provide guidance for the diagnosis and treatment of high-risk females.

In this review, we summarized POF-related genomic variants, transcriptome abnormalities and protein molecular functions according to recent thought-provoking articles and projects. The etiology and molecular basis of POF are highlighted through different prospective cytogenetic constitutions. In addition, some novel concepts challenging the therapeutic options for POF in clinical reproductive trials have also aroused great interest in the management of this issue. We describe the design of several ongoing clinical trials that might propose safe, feasible and effective approaches to improve the diagnosis and treatment of POF.

## Genomic instability involved in POF

**FIGURE 1:**
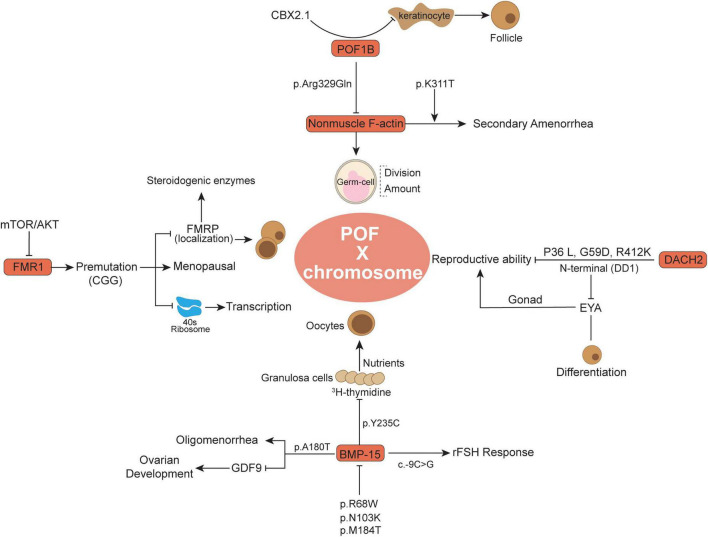
Summary of some X chromosome gene variants and related functions in the POF.

**FIGURE 2:**
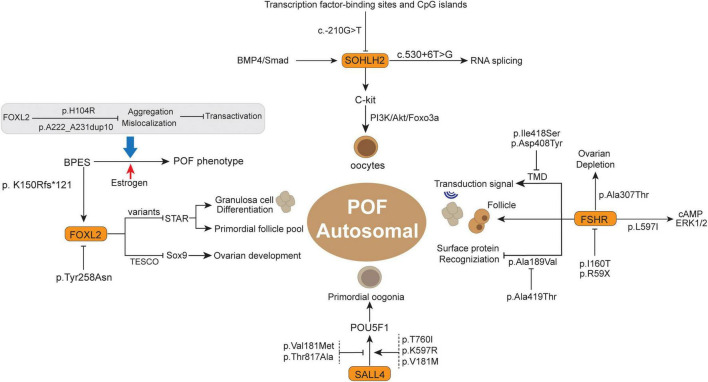
The summary of some autosomal gene variants and related functions in the POF.

Chromosomal gene abnormalities have long been regarded as a cause of POF, but the percentages vary widely among reported series (17). Genetic heterogeneity and disease development have been widely investigated and observed to have a close relationship (18). With the development of sequencing technology, increasing solid evidence suggests that genomic instability underlies the pathogenesis of POF (13). Therefore, as an obstacle to basic ovarian development, key gene variants are particularly harmful to ovarian cells and promote POF (Table 1). Variants located in conserved regions are more likely to influence the inherent gene functions (19) 31871067. Thus, variation-induced protein and cell signaling perturbations might yield severe functional defects in ovarian cell development, thereby causing a negative effect on POF. In this section, we summarize several pivotal genes (X chromosome genes and autosomal genes) and their latest research advances (Figures 1, 2), whose variants warrant strong consideration as pathogenic candidates for POF.

**TABLE 1:** Candidate genetic variants responsible for ovarian disorder and POF.

	Gene	Location	Population	Variations	Protein	Prominent related effects	References
X chromosome genes	POF1B	Exon 10	Lebanese	c.986G > A	p.Arg329Gln	Impair the ability of POF1B to bind non-muscle F-actin, and affected germ cells fenlie in POF	(23)
		Adjacent to exon 10	Chinese	c.932A > C	p.K311T	Impair the capacity of POF1B to bind non-muscle actin filaments and lead to secondary amenorrhea	(26)
		Intron 4	Turkey	c.439-2A > G	Unknown	Change the evolutionally conserved splicing acceptor site	(27)
	BMP-15	Exon 2	Iranian	c.538G > A	p.A180T	reduce mature peptide generation and activity and synergy with growth differentiation factor 9 (GDF9)	(48)
		Exon	Italian	c.704A > G	p.Y235C	Suppressed ovary granulosa cell proliferation and was associated with ovarian dysgenesis.	(43)
		Exon 2	Caucasian	c.202C > T	p.R68W	Influence in BMP15 biological activity and difficult to rescue	(42)
		Exon 2	Iranian	c.309T > G	p.N103K	Shows haploinsufficiency or negative dominance effects and is similar with previous mutation features	(47)
		Exon 1		c.551T > C	p.M184T		
		Promoter	Indian	c.-9C > G	//	Associate with POF and shows the ability to strengthen the assisted reproduction technique response to recombinant FSH	(57, 58)
Autosomes genes	SOHLH2	Promoter	Chinese	c.-210G > T	//	Disturb the SOHLH2 gene expression through interfering the upstream transcriptional regulator recognition	(109, 110)
		Intron 5	Serbian	c.530 + 6T > G	//	Impair RNA splicing and decrease the number of translated proteins in POF	(109)
	FOXL2	Exon	New Zealand	772_1009_T > A	p.Tyr258Asn	Lead to FOXL2 haploinsufficiency	(122)
		//	American	c.448_448delA	p.K150Rfs*121	contribute to POF in the phenotypic variability of BPES	(128)
		Exon	Caucasian	Missense mutation	p.H104R	Cause BPES-induced POF	(130)
				In-frame duplication	p.A222_A231dup10		
		Exon	Mexican	c.76G > T	p.Glu26*	Contribute to FH domain deleted protein and FOXL2 haploinsufficiency	(132)
				c.290delG	p.Gly97Alafs*53		
	SALL4	Exon 2	Chinese	c.541G > A	p.Val181Met	Influence the structure and DNA binding of SALL4	(137)
				c.2449A > G	p.Thr817Ala		
		Exon 2	Chinese	c.2279C > T	p.T760I	Associated with SALL4 protein level and enhanced regulatory activity to downstream POU5F1	(140)
				c.1790A > G	p.K597R		
				c.541G > A	p.V181M		
	FSHR	Exon 7	Finland	566C > T	p.Ala189Val	contribute to the defection of targeting protein in cell surface and serious phenotype of the ovarian failure	(143)
		Exon 6	German	c.479T > C	p.I160T	impair the FSHR expression	(144)
		Exon 10	Asian Indian	c.1253T > G	p.Ile418Ser	Lead to decreased transmembrane signal transduction and follicle retardation	(147)
		Exon 10	Turkish	c.1222G > T	p.Asp408Tyr	Decrease granulosa cell-surface transduction signal and total second messenger cAMP production	(149)
		Exon 10	Chinese	c.1789C > A	p.L597I	Suppress FSH-induced cAMP production and ERK1/2 phosphorylation	(152)
		Exon 10	Asians	c.2039G > A	p.S680N	Serve as a potential genetic biomarker for POF	(156)
		Exon 2	Chinese	c.175C > T	p.R59X	Arrest folliculogenesis, decrease FSHR expression and abolish the granulosa cells response to FSH stimulation	(157)
		Exon 10	Brazil/German	c.919A > G	p.Ala307Thr	Associate with ovary stimulation acceleration, therefore leading to ovarian depletion in POF patients	(144, 160)

## Abnormal X chromosome genes

### The premature ovarian failure 1B (POF1B)

The premature ovarian failure 1B evolutionary novel gene was found only in vertebrates (19) and has been identified by breakpoint mapping of X-autosome translocations (20). Generally, this gene alludes to a specific region following the law of Online Mendelian Inheritance in Man (OMIM) (21). It was previously detected that POF1B (an interrupted gene) in POF patients appeared to be mutated (20). Whole-genome sequencing of a Lebanese POF familial genetic group revealed point mutations localized in POF1B exon 10 characterized by a nucleotide (G > A) at position 1,123 (22). Among these, the protein sequence alteration occurred at 329 amino acid site p. Arg329Gln (c.986 G > A), similar to arginine to glutamine, and the mutant POF1B protein showed a lower ability to bind non-muscle F-actin (Figure 1), thus influencing the POF patients’ germ-cell division (22, 23). Moreover, the POF1B p.Arg329Gln variant influenced the F-actin amount and contributed to the loss of tight junctions in polarized epithelial cells in POF (24). More recently, Zhang et al. demonstrated that there was a novel POF1B missense variant in Chinese POF patients, namely, p.K311T (c. 932A > C), which was adjacent to p.Arg329Gln and was supposed to have similar functions to non-muscle F-actin (25). Recently, in a 21-year-old Chinese POF woman with long-term oligomenorrhea and high sex hormone levels, exome sequencing revealed p.K311T (c.932A > C) variant in POF1B was closely associated with POF (25). The p.K311T was milder than p.Arg329Gln and was more likely to lead to secondary amenorrhea (25). Through comparative analysis with p.Arg329Gln, p. K311T was suspected to damage the capacity of POF1B to bind non-muscle F-actin and lead to secondary amenorrhea (25). Moreover, another variant (c.439-2A > G) in intron 4 of POF1B was observed to be associated with POF, which mainly changes the evolutionally conserved splicing acceptor site (26).

In some keratinocytes, the POF1B expression level was observed to be inversely associated with keratinocyte number and correlated with defects in cell adhesion (27). Keratinocyte-related cytokines might promote the primordial to primary follicle transition (28). As an upstream modulator, CBX2.1 has the ability to stimulate POF1B activation, whereas silencing CBX2.1 significantly downregulated POF1B expression and potentially was associated with POF development (29). CBX2.1 in human sex development has been reported to inhibit the female pathway and is related to female hypoplasia (30). The pathogenic variants of POF1B have been continuously studied (23), and increasing evidence supports the importance of POF1B variants in POF occurrence.

### Bone morphogenetic protein 15 (BMP15)

Bone morphogenetic protein 15 is an X-linked coding gene and is related to primary-stage protein generation from oocytes (31). As a member of the TGF-ß superfamily family, BMP-15 also has significant advances in our understanding of early follicle development and oocyte regulation in mammals (32, 33). BMP-15 was demonstrated to be expressed in oocytes and pituitary cells but to a much lesser extent in other organs, such as the kidney and heart (34). The importance of BMP-15 as a fertility marker in females has attracted great attention because it influences all statuses of oocyte development and embryonic quality, especially the regulation of granulosa generation and ovarian functions (35, 36). Expression of BMP15 in oocytes stimulates granulosa cell growth and inhibits FSH action by suppressing follicle-stimulating hormone receptor (FSHR) expression, which is related to ovulation rate and fertility (37). Compared to other TGFß superfamily members, BMP-15 has undergone rapid evolution and subjective positive selection in mammalian clades, and its important and particular functional role eventually leads to its importance in female fertility (38). Abnormal regulation of BMP-15 might be related to female POF and infertility (39, 40). Recently, attention has been focused on the potential biological impact of BMP15 genomic variation on the POF population. BMP15 variation may predispose to POF and stimulate ovarian defects in cooperation with other alteration features (41).

Notably, Di Pasquale et al. (42) reported that the p. Y235C in the X-linked BMP15 gene in 2 sisters, a heterozygous non-conservative substitution in the pro region, suppressed ovary granulosa cell proliferation and was associated with ovarian dysgenesis. Among these, wild-type BMP15 showed the ability to stimulate ^3^H-thymidine in ovary granulosa cells and was restored at a concentration nearly five-fold higher than that of p. Y235C BMP15 group. Granulosa cells were demonstrated to deliver nutrients and metabolites through gap junctions to oocytes and are involved in oocyte secretion of paracrine signals (43). In addition, BMP15 knockdown female mice have a reduced ovulation rate and therefore show lower fertility (44). BMP15 variation not only influences granulosa cells but also plays an important biological role in the secretion and activation of TGF-β superfamily members in oocytes (45). Moreover, there are two missense alterations (p.R68W and p.A180T) and one insertion (p.262insLeu) were observed in a POF cohort study (33). The p.R68W (c.202C > T) showed a larger and significant influence on BMP15 biological functions, and p.R68W carriers develop POF before the age of 20 years old (41). *In vitro* cotransfection experiments with wild-type cDNA indicated that p.R68W is a deleterious mutation in POF patients, where BMP15 activity is difficult to rescue (41). Moreover, in Iranian POF familial history patients, the detectable p. A180T (c.538G > A) was accompanied by a long history of oligomenorrhea, high FSH and very low anti-Mullerian hormone (AMH) levels, whereas without sonographic abnormal symptoms (46). The p.A180T nucleotide site alteration in exon 2 of BMP15 (named rs104894767) has been recorded in databases. As previously reported, the p. A180T variation was demonstrated to be associated with non-familial POF patients (33). By Sanger sequencing analysis, the POF family members who carry the p.A180T variant presented premature physiological menopause, while healthy members without p. A180T variant presented regular menses (46). Besides, BMP15 with p. A180T variant presented fourfold lower activity than the wild-type, which changes the potential to reduce mature peptide generation and activity and synergy with growth differentiation factor 9 (GDF9) (47). BMP15 protein binds to its closely related paralog GDF9 to form homodimers or heterodimers that regulate many aspects of development by activating transmembrane serine/threonine kinase receptors (42, 48, 49). The interaction between GDF9 and BMP15 mutation is regarded as an important internal factor for ovarian hypofunction in POF patients (47, 48).

Some novel BMP15 mutations were detected in a number of POF patients. In the BMP15 pro-domain region, p.N103K and p.M184T were observed to prevent the protein from binding to mature dimers, consequently leading to decreased BMP15 activity (46, 50). The pro-domain is usually uncoupled during the maturation process and modulates the folding and dimerization of the protein structure (51). BMP15 with p.N103K and p.M184T variant usually showed haploinsufficiency or negative dominance effects and is similar to previous mutation features (46), which is potentially associated with POF symptoms (52–54). Moreover, BMP15 with the c.-9C > G promoter polymorphism presented a functional association with the POF phenotype (49). Although c.-9C > G in 398 PCOS female cohort studies was not significantly associated with disease pathogenesis, it was related to some specific clinical features, such as anovulation and infertility (55). Indeed, c.-9C > G is a frequent variant in BMP15 and has been reported to be functionally associated with POF (56). Due to the high reactivity of the G allele, c.-9C > G also showed the ability to strengthen the assisted reproduction technique response to recombinant FSH (57). Additionally, *in silico* analysis in combination with the BMP15 promoter sequence showed that the -14 to -8 bp region of the BMP15 promoter is the primary regulatory target for the pituitary homebox 1 protein (PITX1) (49), in which two of the active binding regions were validated *in vitro*, namely, prom-G and prom-C constructs. PITX1 was reported to be associated with gonadotroph cell activation and estrogenic signals (58), and the estrus cycle-related estrogen signaling pathway was positively involved in POF traditional Chinese medicine treatment (59). Despite recent findings demonstrating the involvement of BMP15 mutation in POF, further studies elucidating the roles of modulators would lead to a better understanding of the disease pathogenesis.

### Fragile X messenger ribonucleoprotein 1 (FMR1)

Fragile X messenger ribonucleoprotein 1 is an X-linked gene encoding RNA binding protein, and its mutation in the 5′UTR dynamic triplet CGG repeat is related to fragile X syndrome (60–62). The CGG trinucleotide repeat is usual but unique in the region of FMR1 exon 1, which conducts mRNA transcription but not translation into protein amino acids (61). The copies of FMR1 premutation CGG can be expanded to varying degrees and associated with different biological function performance (63). Mutations of FMR1 are usually categorized by the number of CGG trinucleotide repeats: the classic normal is 6-45 CGG repeats in the 5′UTR; FMR1 premutation alleles have 50-200 CGG repeats in the 5′UTR; FMR1 full mutation has an expansion of more than 200 CGG repeats in the 5′UTR; and the intermediate range (also called the gray zone) has an expansion of 45–54 repeats in the FMR1 5′UTR (64, 65). The FMR1 gene is primarily associated with neuro/psychiatric risks, while it appeared to control the function of follicle recruitment and ovarian reserve in recent evidences (63, 66, 67). Moreover, the premutation CGG repeat-related increased FMR1 mRNA transcription was potentially involved in POF pathogenesis compared to the general population, such as 35 and 54 repeats (64). Two independent studies indicated that FMR1 premutation carriers have an earlier average menopausal age and are more susceptible to POF than non-carriers (68, 69). Notably, in familial trait ovarian failure, FMR1 premutation CGG was associated with a large number of ascertained idiopathic POF cases (61). FMR1 premutation expansions are not merely high-risk factors for POF occurrence but are also relevant to cytokine levels, such as serum FSH, in ovarian aging pathological conditions (70, 71).

Recently, the FMR1 premutation aroused great attention to female reproductive fertility, and women with monogenic FMR1 premutation have an equivalent risk of POF to the top 1% of genetic susceptibility (72). Approximately 15–20% of females with an FMR1 premutation develop POF (73). In Europe, the frequency of premutation of FMR1 in POF was higher than that in the general population, and FMR1 sequencing has become part of the recommended monitoring indicators for women with POF (74). However, the FMR1 premutation frequency is relatively lower in Chinese women. In Guo et al. (75) indicated that 0.49% (2 to 379) FMR1 premutation was found in limited sporadic Chinese POF samples, while none were observed in 402 controls. Recently, another case-control study reported that only 1.6% (2 to 124) of FMR1 permutations were observed in Chinese POF patients, and a 0.9% (1 to 111) premutation frequency existed in the control group (76). Studies from a Chinese cohort indicated that the distribution of allele 1 (smaller number of CGG repeats) rather than allele 2 in POI was closely associated with POF occurrence, wherein <26 and ≥29 CGG repeats potentially have a higher risk for reproduction and POF (76, 77). The ectopic expression of FMR1 CGG repeats leading to POF was also validated in a mouse model (78). However, the underlying mechanism of FMR1 CGG repeat-relevant ovarian function modulation remains unclear, including the pathological condition of the reduced ovarian reserve and abnormal follicular and oocyte growth. One hypothesis is that various FMR1 CGG expansions might contribute to transcriptional level changes and lead to ratios of distinct RNA isoforms (61, 64, 79). The above changes potentially decrease the fragile X messenger ribonucleoprotein 1 (FMRP) level or intercellular localization, thereby affecting steroidogenic enzymes and hormonal receptors and ultimately affecting the occurrence of POF (79, 80). The increased CGG repeat in FMR1 has the ability to impede the 40S ribosomal subunit in the process of downstream initiation codon recognition, resulting in translational debility and consequently reducing the FMRP level (80). Generally, during normal folliculogenesis, FMRP is primarily expressed in granulosa cells, which is crucial for oocyte maturation and growth. POF patients with FMR1 CGG repeats, such as 19 < n < 90, have impaired ovarian reserve, and abnormal FMR1 transcript levels related to FMRP influence the process of oocyte and follicular maturation (81, 82). Moreover, in these reproduction-related cells, the intracellular signaling cascade is also involved in FMR1 expression and regulation. Rehnitz et al. (67) reported that inhibition of AKT increases FMR1 expression and decreases FMRP levels, whereas recombinant FSH (rFSH) and mTOR inhibition lead to the opposite phenomenon, indicating that a feedback loop between FMR1/FMRP and the mTOR/AKT signaling cascade interacts during GC proliferation and oocyte maturation. In terms of this, FMRP phosphorylation and activation are thought to be potentially associated with S6K, a downstream molecule of mTOR (83). The FMR1 knockdown mouse exhibited larger ovaries in mass and volume compared to age-matched controls, wherein increased protein levels of Tsc2 and mTOR were detected (84). As previously reported, FMR1 expression influenced cholesterol and steroid hormone generation and impaired ovary responses to hormonal stimulation and growth (78, 82), while the mTOR pathway was involved in ovarian development and enlarged ovaries histologically with precocious follicular development (84, 85). On the other hand, an *in vivo* experiment in mice observed that permutation transcripts were closely associated with mitochondrial and ovarian abnormalities (86). mTOR is closely related to the metabolic process of mitochondria in ovarian cells (87), and inhibition of mTOR significantly improved follicular development and endocrine functions of the ovaries, thereby extending reproductive aging and premature aging of POF mice (88). These observations suggested that abnormal FMR1 transcription potentially causes POF, but it remains unclear which process plays a prior role in disease initiation.

### DACH2

The Dachshund (DACH) gene family is known as a transcriptional cofactor on the basis of highly conserved protein interaction domains, while the DACH genomic mutation was observed to be closely associated with poor female reproductive tract development, namely, DACH1 and DACH2 (89). The DACH gene was first described in Drosophila, encoding nuclear proteins involved in different organs, such as eyes, limbs, and genital discs, which are required for the genital development of male and female phenotypes (90, 91). In mammals, the female and male reproductive tracts develop from the Müllerian duct (MD) and Wolffian duct (WD), respectively, wherein the WD regresses, retains the MD and then differentiates into the oviduct, uterus, and cervix (92, 93). Both DACH1 and DACH2 were observed in MD, and DACH1/DACH2 double mutation is associated with female reproductive tract development retrogression (89). In addition, DACH2 is expressed in human ovarian tissue and contributes to some ovarian malignant diseases, and variants in DACH2 may be associated with the POF phenotype (20, 94). Specifically, DACH2 has been implicated in human POF syndrome, where the genomic alteration of DACH2 was demonstrated to hinder the correct process of ovarian follicle differentiation (95).

The DACH2 gene in Xq21 is located 700 KB distal to POF1B (96–98). The genomic mutation analysis of the DACH2 coding region observed that missense mutations of three single nucleotide polymorphisms (SNPs) (P36 L, G59D, and R412K), non-conservative amino acid substitutions, were frequently present in POF patients compared to the control group (20). These three mutations were proven to be specific in the DACH2 subfamily rather than DACH1, and they occur in the N-terminal region of the DACH2 protein close to the DD1 domain (amino acids 66–162). The DD1 domain at the N-terminus was demonstrated to be involved in DNA binding and EYA protein interaction; thus, DACH1 genomic mutation potentially influenced its signaling biological functions (99, 100). In an animal model, the EYA gene plays an important role in mammalian gonad development, whose response to estrogen-related receptors will directly influence reproduction (101). Therefore, the interaction between DACH2 alteration and EYA protein might increase the risk of POF by affecting the biological process of ovarian follicle differentiation (20, 102). Moreover, another study indicated that genomic mutation was specific to one POF patient, which occurred in the third intron of DACH2 with a C to T transition downstream of Exon_3 (103). On the basis of their results, this variant was not within the splice or receptor sequences and was unlikely to influence the DACH2 level, and there is no clear evidence to support its direct function in POF pathology. Strengthening the screening of more candidate disease-causing mutation sites, including exons and introns, is helpful for the early diagnosis and prevention of POF. Generally, the nucleotide translocation might disrupt the transcription of itself or nearby genes by affecting the cis/trans-acting regulatory factors from the transcription units and consequently deleterious changes in the expression and protein level (89, 104). Nevertheless, there is evidence indicating that DACH2 knockout mice are viable and fertile, which is reminiscent of null mutations of DACH2 that might independently affect the reproductive phenotypes of POF patients (105).

## Autosomal gene abnormalities

### SOHLH2

The transcriptional regulator SOHLH2 was observed to be preferentially expressed in oocytes of immature ovaries and has been deemed a critical regulator for early germ cell development (106). The SOHLH2-silenced mouse model showed infertility and atrophied ovaries devoid of follicles (107). Previously, Qin et al. (108) indicated that SOHLH2 variants were implicated in primary POF, which exhibited several non-synonymous mutations in POF patients compared to normal controls. For example, p.Glu79Lys (c.235G > A) and p.Glu105Gly (c.314A > G) have high conservation among mammalian potential to be pathogenic for Chinese POF patients. In addition, the SOHLH2 promoter variant c.-210G > T was observed in a Chinese POF patient and is located in a region with transcription factor-binding sites and CpG islands (Figure 2) (108, 109). This variation may potentially disturb SOHLH2 gene expression by interfering with upstream transcriptional regulator recognition (109). Notably, the loss of SOHLH2 expression contributes to the rapid loss of oocytes, as well as increased oocyte apoptosis (110). In this respect, SOHLH2 has been regarded as the downstream target of the BMP4/Smad signaling pathway in the survival and apoptosis of oocytes (111). Moreover, SOHLH2 could bind to the C-kit promoter, act as a positive transcriptional regulator for C-kit and modulate the C-kit/PI3K/Akt/Foxo3a cascade in oocytes, which is directly associated with oocyte survival and follicle development in POF (111, 112). Furthermore, there is another variant (c.530 + 6T > G) in introns that is supposed to affect RNA splicing, thereby decreasing the number of translated proteins in POF (108).

### FOXL2

Forkhead box L2 (FOXL2) belongs to the winged helix/forkhead transcription factor family, containing a 110 amino acid DNA-binding domain, which is associated with protein mislocation, aggregation, and intranuclear mobility, including ovarian development and postpartum recovery (113). FOXL2, the earliest recognized ovarian differentiation marker in mammals, was observed in the granulosa cells of the ovary as well as the mesenchyme of the developing eyelids (114–117). FOXL2 might be a pivotal modulator of ovarian development and eyelid formation. Previous studies also concluded that the FOXL2 variant results in a relatively mild blepharophimosis-ptosis-epicanthus inversus syndrome (BPES) phenotype (118), which is a rare autosomal dominant genetic developmental disorder in the eyelids and ovary (114). BPES has emerged to have two subtypes: type I is associated with POF, and type 2 has no systemic associations (119), wherein the different amino acid site alterations will lead to the different subtypes (120).

Previously, the FOXL2 variants A221-A230del and p.Tyr258Asn (772_1009_T > A) were observed in POF patients from the New Zealand and Slovenia cohorts, respectively (121). The non-conservative substitution of 772_1009_T > A might lead to FOXL2 haploinsufficiency, thus promoting POF development (121). After that, some studies indicated that FOXL2 variants of p.Gly187Asp and c.627delT favor the implication of FOXL2 variants in POF, and more systematic genetic screening of FOXL2 variants is important in POF premature diagnosis and hormonal replacement therapy (122, 123). Recently, several FOXL2 variants were reported to be associated with a severe BPES phenotype, such as p.Arg103Cys (c.307C > T), p.His104Pro (c.311A > C), p.Ser107Asn (c.320G > A), and p.Phe112Tyr (c.335T > A) (124). Of note, these variants potentially damage the function of STAR and OSR2 protein by abolishing transcriptional activity (124), and STAR is important for differentiation of granulosa cells, where the FOXL2 variant will lose suppression for the STAR promoter, thereby accelerating differentiation of granulosa cells and secondary depletion of the primordial follicle pool (125, 126). In addition, the FOXL2 heterozygous deletion variant p.K150Rfs*121 (c.448_448delA) and its coexisting gene BMP15 act synergistically and contribute to POF in the phenotypic variability of BPES, and this variant leads to polyalanine deletion and truncated protein at 269 amino acids (aa) (127, 128). Herein, the digenic inheritance of the FOXL2 variant and its related effector potentially contribute to BPES-related ovarian function impairment and POF 25988799. Moreover, two variants, p.H104R and p.A222_A231dup10 in Caucasian FOXL2 leads to mislocalization and aggregation, thus impairing transactivation, which could cause BPES-induced POF (129). In one case, estrogen treatment successfully improved menarche and secondary sexual characteristics, which showed promising therapeutic potential in the reproductive outcomes of BPES-related POF patients (130). In a Latin American cohort, two FOXL2 variants (c.76G > T and c.290delG) were also deemed pathogenic factors for POF patients, wherein the former contributes to FH domain deleted protein and FOXL2 haploinsufficiency, while the latter mechanism is still unclear (131). Generally, the FOXL2 protein acts as a transcription factor for some important molecules in ovarian maintenance and function, such as binding to TESCO to suppress Sox9 expression in ovaries, ultimately contributing to the development of ovaries (117). The polyalanine tract deletion/expansion-related variant is likely to abolish FOXL2 protein function and thereby abrogate the above process in ovarian maintenance (132), where the polyalanine tract deletion variant is more likely related to BPES type I (with POF) (121, 133). Meanwhile, there are many variants present in unaffected POF family members that have not been confirmed to be pathogenic variants. Identification of FOXL2 variants is critical for suspected BPES and/or POF evaluation as well as prevention in due course.

### SALL4

Spalt-like transcription factor 4 (SALL4), a zinc finger transcription factor expressed in murine oocytes, binds to POU5F1 and regulates its expression (134, 135). Previously, genomic sequencing of 100 Han Chinese POF women showed that the SALL4 variants p.Val181Met (c.541G > A) and p.Thr817Ala (c.2449A > G) potentially influences the structure and DNA binding of SALL4 and is associated with POF development (136). The pathogenesis of the SALL4 variants might be achieved by abolishing the recognition of the downstream POU5F1 gene and suppressing its expression (135, 137), ultimately affecting primordial oogonia development and triggering POF (138). Recently, another POF study in 50 Han Chinese individuals through whole-exome sequencing (WES) analysis discovered several novel variants in POF patients, including p.T760I (c.2279C > T) and p.K597R (c.1790A > G), as well as the verified variant p.V181M (c.541G > A) (139). Intriguingly, *in vitro* functional experiments showed that these variants were positively associated with the SALL4 protein level and enhanced regulatory activity to downstream POU5F1. According to this phenomenon, posttranslational regulation of SALL4 protein levels might be the molecular mechanism underlying POI (139). Taken together, the above observations suggested that SALL4 variants are closely associated with POF development, whereas the different variation types of SALL4 might present different manifestations of SALL4 activity and phenotypic variability.

### FSHR

Follicle-stimulating hormone receptor has a pivotal role in recognizing FSH, thereby controlling granulosa cells of the ovary and female reproduction (140). FSHR variants have been identified in women with hypergonadotropic POF symptoms, especially inactivating mutation subtypes (141). Doherty et al. (142) reported that inactivating extracellular FSHR variants existed in the ligand recognition region of the receptor, namely, p.Ala189Val (566C > T), whose occurrence contributed to the defect of targeting protein in the cell surface and a more serious phenotype of ovarian failure. Interestingly, another transmembrane variant, p.Ala419Thr, of FSHR reduced symptoms when it compounded with p.Ala189Val, which was potentially due to the higher residual activity retained by the transmembrane variant and neutralizing p.Ala189Val variant inactivating functions (142). Notably, Ledig et al. (143) observed that p. I160T (c.479T > C) is an inactivation variant in FSHR and is associated with POF, which will impair FSHR expression on the cell surface. They simultaneously indicated that p.I160T variant-induced FSHR inhibition potentially further enhanced the BMP15 variant (p.A180T)-related granular cells decrease in the developing follicle (143). In addition, the interaction between FSHR SNP rs6166 and CYP19A1 SNP rs4646/rs10046 was demonstrated to be involved in POF development by regulating folliculogenesis (144). Thus, synergistic effects of digenic variants might promote POF development to a considerable extent.

Moreover, there are still many FSHR variants that have been demonstrated to have independent pathogenic roles in POF. FSHR is important in human reproduction and was proven to be the first single gene to cause POF (145). In an Asian Indian descent family, an inactivating pathogenic variant p.Ile418Ser (c.1253T > G) in FSHR was detected in POF patients (146). The p.Ile418Ser (c.1253T > G) occurred in exon 10 of FSHR and impaired the transmembrane helix of the FSHR protein, which led to decreased transmembrane signal transduction and follicle retardation (146). Based on characteristics in the FSHR helix transmembrane domain (TMD) and highly conserved across species (147), inactivated p.Ile418Ser variant in FSHR was supposed to cause POF. In the second TMD of FSHR, another inactivating variant, p.Asp408Tyr (c.1222G > T) has been observed in two Turkish POF patients, which was also accompanied by decreased granulosa cell-surface transduction signal and total second messenger cAMP production (148). The lack of sufficient FSHR expression and function in ovarian granulosa cells are unable to promote follicle maturation and ovulation, despite high levels of FSH stimulation (149, 150). Recently, a large cohort study in Han Chinese with more than 190 POF patients and normal controls detected some novel variants. The p. M265 V (c.793A > G) and p.L597I (c.1789C > A) variants exclusively existed in Chinese POF patients, former located in extracellular domain (ECD) and later in TMD, wherein the p.L597I can suppress FSH-induced cAMP production and ERK1/2 phosphorylation, thereby stimulating the POF phenotype (151). Generally, the FSHR variant in ECD impaired trafficking and cell surface expression, while the variant in TMD was characterized by abolished signal transduction (152, 153). However, the p.L597I decreased FSHR expression in the membrane (151), which is similar to another inactivating variant p.A575V (TMD) manifestation in primary amenorrhea patients (154). In addition, another POF-associated FSHR variant p.S680N (c.2039G > A) was specifically proven in Asian people rather than other ethnicities and is supposed to serve as a potential genetic biomarker for POF in Asians (155). Through comparison between a Chinese POF family and 192 control women, Liu et al. (156) demonstrated that a novel FSHR variant p.R59X (c.175C > T) in exon 2 was causative for POF by arresting folliculogenesis. The p.R59X is positively related to decreased FSHR expression and deemed a loss-of-function variant. Additionally, p.R59X variant will abolish the granulosa cell response to FSH stimulation, mainly due to the truncated FSHR protein-related ECD and TMD function region absence (156). It is thus clear that inactivation of FSHR variants will impair FSHR functions and follicular development, thereby leading to POF (157). Further study should focus on pharmacological and assisted reproductive treatments targeting disrupted FSHR.

Patients with specific FSHR variants, such as p.Ala307Thr polymorphism in Brazilian patients, while it was not associated with ovarian endocrine variables or clinical ultrasonographic findings (158). Moreover, an expanded comparative sample identified that p.Ala307Thr is more frequent in POF patients than in controls (159). Among these, increased FSH levels in p.Ala307Thr carrier might be associated with ovary stimulation acceleration, therefore leading to ovarian depletion in POF patients (159). Notably, in another study, the p.Ala307Thr variant has also been identified in 3 German patients with POF (143). In terms of this, menstrual dysfunction precedes the initiation of amenorrhea, such as oligomenorrhea, transient amenorrhea and short cycles, which might be a special signature for ovarian failure and are regarded as “prodromal POF” (160). In terms of this, future investigations are needed to explore the potential roles of other FSHR variants in the development of POF.

### Dysregulated gene expression in POF

**FIGURE 3:**
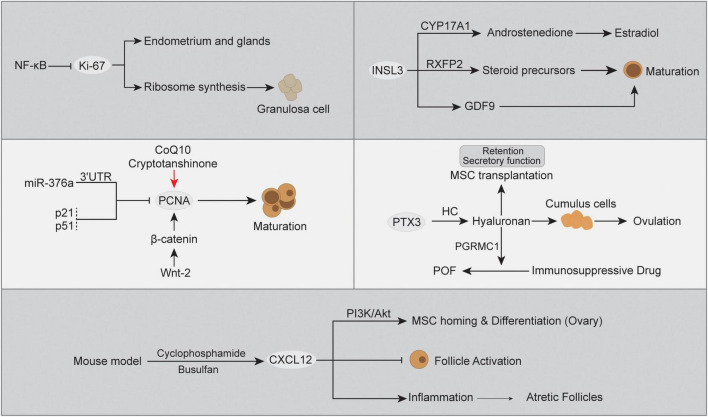
The abnormal gene expression and relevant cascades in POF.

Apart from genomic alterations, abnormal expression of the transcriptome caused by multiple factors also has a great impact on POF. Ovarian insufficiency is a continuum of impaired ovarian function or ovarian aging, which is always accompanied by signature marker gene expression abnormalities (161). In POF, there are three consecutive but progressive stages, occult, biochemical, and overt ovarian failure (162), and gene expression changes with pathogenesis can provide better evidence for clinical diagnosis (163, 164). The monitoring of gene expression has been widely used in the discovery of disease biomarkers or therapeutic targets (165). However, to our knowledge, the molecular mechanism of POF has not yet been clarified. Below, we summarize the dysregulated expression of POF-related genes identified by recent studies, including Ki-67, proliferating cell nuclear antigen (PCNA), chemokine (C-X-C motif) ligand 12 gene (CXCL12), insulin-like peptide 3 (INSL3), and PTX3 (Figure 3).

### Ki-67

Ki-67 is expressed in proliferating cells, and its protein is associated with different nuclear domains, whereas the Ki-67 protein was detected in mouse oocytes rather than mature sperm (166, 167). Ki-67 is a DNA-binding protein expressed in all active cell cycle stages and can be used as a marker for cell proliferation (168, 169). Herein, Ki-67 expression has been regarded as the signature for ovarian tissue transplantation activity and was used to evaluate transplantation-associated follicle dynamics, where the higher Ki-67 expression in ovarian granulosa cells indicated an increase in activated growing/primordial follicles (170). Moreover, an *in vivo* POF model indicated that the ovarian proliferation index can be evaluated by Ki-67, which is expressed in epithelial cells of the endometrium and glands (171). Additionally, lower expression of Ki-67 was detected in the POF group than in the control group. In contrast, ovarian tissue with higher FSH levels showed morphologically normal follicles and significantly decreased Ki-67 expression (172, 173), and gonadotropin inhibited mitosis in granulosa cells early in the periovulatory interval (174). When follicular atresia occurs, the mutual intensive interaction between granulosa cell death and oocytes plays an indispensable role (175). Moreover, the inhibition of NF-κB signaling reversed the loss of Ki-67 expression in granulosa cells (176). A previous study indicated that CYP19A1 expression and estradiol secretion in human ovarian granulosa cells were directly modulated by NF-κB signaling (177), and these two elements and Ki-67 expression have served as indicators in ovarian disease estrogen treatment (178). The intervention of Ki-67 expression will directly promote ribosome synthesis during cell division, which is necessary for ovarian granulosa cell proliferation (179). This evidence supports it as a useful feature to better understand ovarian granulosa cells and follicles, as well as abnormalities in human POF.

### PCNA

Proliferating cell nuclear antigen (PCNA) is a key factor for DNA replication and cell cycling, which can be used as an indicator of ovarian cell proliferation and define the extent of departure from Strzalka and Ziemienowicz (180), Thomas et al. (181), Muskhelishvili et al. (182). Through immunohistochemical detection, PCNA has been proven to distinguish follicles with different maturation statuses, where PCNA expression is significant in oocytes, granulosa cells and growing follicles and acts as a primary regulator (183, 184). However, atretic follicles undergoing atresia were negative for PCNA expression (185). In a secondary POF model, the mRNA expression of PCNA was significantly decreased in ovarian tissue compared with healthy controls (186). Moreover, coenzyme Q10 (CoQ10) and/or cryptotanshinone treatment promoted primary follicle and granulosa PCNA expression and improved ovarian injury in POF (186, 187). A previous study indicated that miR-376a influenced primordial follicle assembly and oocyte apoptosis by binding to the mRNA 3’ untranslated region (3’-UTR), thereby inhibiting PCNA expression (188). The microRNA modulatory effect on gene expression has been proven to be associated with POF in a mouse model (189). In POF, increased p53 might disturb oocyte quality and damage ovarian functions (190), mainly through a posttranslational mechanism (191). In this respect, the cyclin-dependent kinase suppressor protein p21 is a key downstream target for p53, where p53 boosts downstream effector expression by elevating nascent RNA amounts (192, 193). Meanwhile, p21 is known as a dual inhibitor for both cyclin-dependent kinases and PCNA, and p21 has the ability to displace chromatin-bound PCNA to interfere with S phase (194, 195). Not surprisingly, the upregulated p53 expression and enhanced p21 transcription simultaneously existed in POF ovarian tissues and were positively associated with disease development (196). The reduction in PCNA will lose the balance between apoptosis and fail to ensure successful follicle development and protect follicular growth tissue homeostasis (197). In addition, another regulator of follicular growth is the Wnt signaling pathway, which is important for pregranulosa cell transition during the period of primordial follicle activation (198). Notably, significant expression of Wnt-2 in all stages of follicles was positively associated with PCNA and induced granulosa cell proliferation (199). A study in human cumulus cells demonstrated that Wnt-2 recognizes its receptor FZD9 to regulate the formation of β-catenin and E-cadherin (200). The knockdown of β-catenin in granulosa cells inhibited PCNA expression but did not affect Wnt-2 expression (199). Herein, the Wnt-2/β-catenin cascade controls diverse ovarian developmental processes and has the potential to modulate PCNA expression in POF (201).

### CXCL12

Chemokine (C-X-C motif) ligand 12 gene, also known as stromal cell-derived factor (SDF-1), interacts with its receptor CXCR4 and plays an essential role in primordial germ cell (PGC) migration, proliferation, and survival (202). Moreover, there is evidence indicating that the expression of CXCL12/CXCR4 was increased in a POF mouse model and was negatively associated with primordial-to-primary follicle transition (203). In the Chinese Han population, CXCL12 polymorphism-related CXCL12 expression and high CXCL12 protein levels were supposed to be associated with POF and have the potential to be candidate biomarkers (204). A high level of CXCL12 expression has been deemed a potentiator for primordial follicle densities and smaller follicle sizes due to chemokine and receptor interactions, and increased CXCL12 expression inhibits follicle activation and ovarian functions (205). However, the detailed CXCL12 regulation mechanisms in the pathogenesis of POF are still unclear. In terms of inflammation, POF-related atretic follicles and granulosa cells are usually characterized by an inflammatory response involving leukocytes and their secretory inflammatory factors (206, 207). Among them, the interaction between CXCL12 and its receptor CXCR4 was demonstrated to participate in physiological inflammatory processes, including follicular dysregulation (208, 209). In line with this, the cyclophosphamide- and busulfan-induced POF mouse model presented a positive correlation between CXCL12/CXCR4 protein expression and the inflammatory response compared to the normal control, namely, increased proinflammatory cytokines (IL-6, IL-8, and TNF-α) and decreased anti-inflammatory cytokines (IL-10) (203). On the other hand, the chemotaxis role of the CXCL12/CXCR4 axis in mesenchymal stem cell (MSC) transplantation also attracted great interest in POF therapy. Ling et al. (210) reported that CXCL12 can induce CXCR4-expressing MSC migration and homing in the ovaries of POF mice, and blocking the CXCL12/CXCR4 axis significantly reduced MSC homing to ovaries and reduced their therapeutic efficacy in POF. As in previous studies, the CXCL12/CXCR4 axis might be a prerequisite for MSC homing, where CXCL12 acts as a chemoattractant molecule to guide CXCR4^+^ MSC directional migration (211–213). In this process, the activated PI3K/Akt signaling pathway was implicated in the CXCL12-CXCR4 interaction as a downstream factor and is thought to be involved in POF (210, 214). The CXCL12/CXCR4 pathway is known as an upstream switch for Akt phosphorylation, which in turn modulates various biological effects, such as cell migration, chemotaxis and adhesion (215). The binding of CXCL12 to CXCR4 was accompanied by PI3K/Akt signaling activation, while CXCL12/CXCR4 inhibition synchronously inhibited PI3K/Akt signaling, which directly decreased the MSC transplant treatment effect in the POF model (210). The higher homing rate and survival of MSCs to the ovary in individuals will reduce depletion of germline stem cells and increase the therapeutic efficacy for POF (216). Moreover, activation of PI3K/Akt signaling has been reported to promote MSC differentiation into endothelial cells (217, 218) and is positively associated with granulosa cells and follicle proliferation in therapy for POF (219–222). In contrast, suppressed PI3K/AKT signaling probably contributed to large-scale oocyte loss and more serious POF (223). Moreover, PI3K/Akt signaling in MSCs post-transplant also showed the ability to increase the Th17/Tc17 and Th17/Treg ratios to improve the inflammatory immune environment, thereby promoting the recovery of ovarian function in POF (222). Together, the balance of CXCL12 expression is important in POF development, while the pros and cons of mechanisms should be further investigated.

### INSL3

Insulin-like peptide 3 (INSL3) is a member of the relaxin family of neohormones, which is thought to be specific for mammalian traits with respect to reproduction (224). INSL3 is mainly produced by interna cells of the growing antral follicle and is recognized by the specific receptor RXFP2, which modulates the synthesis of the steroid precursor androstenedione (225). In the follicle, INSL3 expression and activation orchestrate the generation of steroid precursors and rostenedione and promote estradiol release in granulosa cells (225, 226). Of note, INSL3 expression was absent in preantral, atretic follicles, granulosa cells or oocytes (227, 228), while RXFP2 was expressed in mammalian oocytes and was associated with oocyte maturation (229, 230). Moreover, INSL3 can also be secreted into the circulation and detected in serum, with a level of immunofluorescence detection of ∼100 pg/ml in women, by which we can monitor the growth of antral follicles (231). As described above, INSL3 expression is consequently increased in polycystic ovary syndrome (PCOS) and decreased in females with POF, which might be a valuable biomarker for POF patients. Importantly, the effect of high INSL3 expression on follicle development might be attributed to the stimulation of GDF9 in oocytes, and the inhibition of GDF9 specifically blocks the INSL3 growth-stimulating effect (232). Moreover, INSL3 from theca cells induces the generation of the enzyme 17α-hydroxylase (CYP17A1) in the same cell, which can modulate the production of the follicular steroid precursor and androstenedione from pregnenolone or progesterone (225). Additionally, released androstenedione will be absorbed by granulosa cells and act as a precursor for estrogens (estrone, estradiol). In the development of POF, the serum INSL3 level was observed to be continuously decreased and showed a strong negative association with FSH (233). Thus, INSL3 might be a promising new specific biomarker for POF progression.

### PTX3

PTX3 is a glycoprotein with two structural domains: one is in the C-terminal region and homology with C-reactive protein (CRP)/serum amyloid P component (SAP), and the other is a unique N-terminal domain without homology (234). PTX3 is specifically expressed in the ovarian cycle and has multifunctional properties under different conditions. Of note, PTX3 was demonstrated to be expressed in cumulus cells, a subtype of granulosa cells surrounding oocytes (235). Cumulus cells are special and have different fates compared to other granulosa cells, such as facilitating oocyte release and fertilization (236). In a mouse model, PTX3 expression is significantly increased in cumulus cells before ovulation and is associated with cumulus matrix formation, and PTX3 blockade in mice results in infertility (237). Due to the specific recognition of heavy chains (HC) to the PTX3 N-terminal domain and its covalent linkage to hyaluronan (HA) polymers (238), PTX3 indirectly influences the HA biological context in cumulus cells, which can be reversed by PTX3 blockade (239). Herein, PTX3 might modulate the HA cascade via the interaction between the HC (240). Notably, HA was reported to be an excellent cell scaffold for MSC transplantation in POF, which not only promotes cell secretory function but also prolongs the retention of MSCs to improve therapeutic efficiency (241, 242). Moreover, HA also protects ovarian function in an immunosuppressive drug-induced POF mouse model, where HA improves granulosa cell damage, estradiol concentration, and the number of follicles (243). In ovarian granulosa cells, HA activation potentially increased progesterone receptor membrane component 1 (PGRMC1) expression, thereby preventing abnormal granulosa cell apoptosis and follicle loss in POF (244). Together, these data indicate that altered expression of PTX3 might influence the POF ovarian microenvironment and cell functions, likely ameliorating proliferation, damage, and hormone levels.

### POF relevant clinical trials

Although the POF incidence rate only accounts for 1% of women under the age of 40 (245), monitoring and management show potential to prevent POF from devastating outcomes (246). POF, a term that appropriately describes the end-stage of premature ovarian insufficiency, is typically diagnosed when amenorrhea combined with high gonadotrophins and hypoestrogenemia, wherein some adolescent patients have follicular depletion or insult to the ovary and present with delayed puberty or amenorrhea (247, 248). Current therapeutic management of POF includes psychosocial support, hormone replacement therapy, and fertility management (249). The diverse etiologies of POF, such as genetics, immune disorders, and microenvironmental dysregulation, have also attracted great attention in the treatment of POF (250). Interestingly, providing more information regarding the etiology, diagnosis, and treatment of POF in adolescents or high-risk populations will provide new insights into preventing disease development, along with the development of more sensitive markers. Below, we will present the latest clinical trials on the diagnosis and treatment of POF.

### Diagnosis

The POF is serious clinical disease with high FSH, low hormonal and ovarian failure. Women of reproductive age diagnosed with POF or premature ovarian failure according to ESHRE criteria are usually based on a sinus follicle count (AFC), AMH, and early follicular serum FSH levels (251). The premature ovarian insufficiency is usually accompanied with anovulatory cycles leading to abnormal uterine bleeding (AUB), it is important to identify the pathogenies for irregular cycles such as FSH, LH, and estradiol measurements (252). The FSH levels of premature ovarian insufficiency women are typically higher than 25 mIU/ml (253), but precise cutoff levels have not been determined. Some patients with premature ovarian insufficiency symptoms show low FSH levels compared with above standard, while FSH > 40 mIU/ml was deemed as POF (254, 255). In addition, simultaneous measurement of upregulated basal luteinizing hormone (LH) levels is helpful to determine whether a high FSH level is associated with ovulation (254). In clinical practice, more stable and detectable indicators are urgently needed. A clinical trial using a combination of FSH stimulation and transvaginal ultrasound examination developed a method for the precisely detection of POF (NCT00006156). Among these interventions, FSH intervention in normal ovaries showed a significant stimulation in serum inhibin B, and ultrasound examination will further define parameters that could improve the earlier diagnosis of POF (256). Moreover, ovary ultrasound characteristics appear to predict ovarian activity, where ovarian volume/area and follicle count are associated with the age of menopause and primordial follicles in POF patients (257, 258). Some clinical trials have been established for endogenous etiological substances, such as the etiological elements endocan, sFlt-1, PIGF, and niacin, in POF patients (NCT03924648, NCT03932877 and NCT04641624). The above prospective studies included POF patients and normal controls with some potential molecular analysis, and blood was obtained at the early follicular phase of the menstrual cycle. These molecular functions are not well studied in the POF mechanism and diagnosis, whereas they are significant in follicle development and POF population (259–261). These works underscore the idea that additional attempts are needed to understand the impact of POF discrete microenvironments and potential diagnostic markers.

### Therapeutic strategies

A randomized clinical trial from South Valley University proposed a hypothesis by using filgrastim to recover ovary functions, with 10 participants from 16 to 40 years (NCT02783937). Filgrastim is a granulocyte-colony stimulating factor (G-CSF) and has been approved to stimulate peripheral blood stem cell numbers (262) and potentially improve ovarian follicle formation (263). In ovarian failure, there are still some residual very small embryonic-like stem cells (VSELs) that serve as a backup pool for mature stem cells and are mobilized under stress conditions, which might be involved in gonadal rescue after exogenous/endogenous stimuli (264, 265). Filgrastim treatment in combination with stem cell studies in a mouse model showed promising results in the recovery of oogenesis and reproductive capacity (266, 267); thus, further clinical studies in humans potentially promote more appropriate treatment for POF. Recently, another clinical trial with 150 participants used the natural plant antitoxin resveratrol to target the NOX/ROS cascade and improve oxidative stress in POF patients (NCT05410093). NOX function in oxidative damage is harmful to ovarian function and structure (268). Moreover, resveratrol exhibited estrogen-like effects and showed great potential in estrogen deficiency-related osteoporosis, as well as increasing ovarian serum estrogen (269). Estrogen upregulation will improve the POF clinical presentation. In this respect, some chemotherapy-induced POF was potentially prevented by the gonadotropin-releasing hormone agonist goserelin (270). The application of goserelin is associated with amelioration of ovarian reserve markers such as AMH, estradiol and FSH, as well as improving ovarian function (271). Nevertheless, further exploration of goserelin function in chemotherapy-induced POF through ovarian function biomarkers are still ongoing (NCT04536467). On the other hand, many trials have focused on systemic balance regulation in POF treatment. Zhang et al. (272) demonstrated that Kuntai capsule, a traditional Chinese medicine, has been widely used for the clinical treatment of menopausal syndrome and showed the ability to improve damaged ovarian function. By improving atretic follicles, AMH expression, the antioxidant pathway, and the Bcl-2/Bax-related apoptotic pathway, Kuntai capsule presented therapeutic potential in the symptoms caused by ovarian failure and ovarian endocrine function recovery. In addition, another POF mouse model demonstrated that Kuntai capsules might inhibit PI3K/AKT/mTOR signaling by decreasing the phosphorylation of pathway protein members, as well as recovering AMH, FSH, and estradiol, ultimately improving ovarian function and protecting reproductive capacity in POF (273). More recently, a random clinical trial with 120 participants was established, which aimed at the efficacy and safety of the Kuntai capsule in POF patients and first used the Kupperman score to evaluate the therapeutic efficacy of the Kuntai capsule (NCT05021094). Another traditional Chinese medicine (HuYang YangKun Formula) was also put forward for POF treatment (NCT02794948), whose systematic regulatory effect significantly improves the ovarian function of POF, such as AMH, FSH, follicle number, TGF-β/TAK1 signaling, and JAK2/STAT3 signaling (274, 275). The ongoing clinical trial is exploring more effective strategies to address these issues so that we can begin to provide etiology-based management for POF-affected women.

## Conclusion and prospective

Despite the increase in emerging research, the underlying mechanisms of POF remain poorly understood due to its variable etiology and complex microenvironment. We summarized the recent dynamic changes in POF that are partly important relevant biomarkers in ovarian development by animal and clinical data, which provided essential evidence to confirm the heterogeneity of genomic variants, gene expression and relevant proteins in POF phenotype and etiology. This review evaluated the role of the above important factor status and pathogenic mechanisms in POF, as well as the downstream and downstream consequences. Future genetic studies should involve different ethnic groups and larger sample sizes to promote the understanding of underlying genetic mechanisms in POF. Additionally, effective diagnosis and management necessitate more reliable biomarker targets in the early point POF, wherein the residual ovarian functions may offer an invaluable chance to intervene early. Currently, the therapeutic options for ovarian functional decline, either physiologically or pathologically, are limited. Established clinical trials provide new insights into developing strategies for ovarian resumption and fertility improvement in POF patients. However, the limitations of current genomic studies have restricted the clinical exploration of POF therapy. Therefore, exploring gene-based effective diagnosis and treatment strategies will be beneficial for the physical, mental, and reproductive health of POF patients.

## Author contributions

XY: original draft and visualization preparation. LY: supervision and concepts of the manuscript. Both authors read and agreed to the published version of the manuscript.
